# Evaluating the quality of multiple automatically produced segmentation variants of the prostate on Magnetic Resonance Imaging scans for brachytherapy

**DOI:** 10.1016/j.phro.2025.100852

**Published:** 2025-10-15

**Authors:** Arkadiy Dushatskiy, Peter A.N. Bosman, Karel A. Hinnen, Jan Wiersma, Henrike Westerveld, Bradley R. Pieters, Tanja Alderliesten

**Affiliations:** a Centrum Wiskunde & Informatica, Evolutionary Intelligence, Amsterdam, the Netherlands; b Amsterdam University Medical Centers, University of Amsterdam, Radiation Oncology, Amsterdam, the Netherlands; c Erasmus Medical Center Cancer Institute, Radiotherapy, Rotterdam, the Netherlands; d Leiden University Medical Center, Radiation Oncology, Leiden, the Netherlands; e Delft University of Technology, Faculty of Electrical Engineering, Mathematics and Computer Science, Algorithmics group, Delft, the Netherlands; f Cancer Center Amsterdam, Imaging and Biomarkers, the Netherlands

**Keywords:** Deep learning, Prostate, Segmentation, Brachytherapy, MRI, Observer variation

## Abstract

**Background and Purpose:**

Recently, we introduced a novel Deep Learning (DL) based (semi-)automatic method for medical image segmentation. Unlike classical DL segmentation methods, it produces multiple segmentation variants (reflecting the variation of manual segmentations) instead of just one. Potentially, with this approach, there is a higher chance that a clinician prefers one of automatically produced segmentation variants. This work focuses on evaluating this method on prostate segmentation in MRI scans used for brachytherapy and investigating its potential clinical usefulness.

**Materials and Methods:**

Three experienced radiation oncologists graded (per-slice and per-scan) segmentations produced by our method, reference segmentations (manually created and used for brachytherapy treatment planning) and segmentations produced by a classical DL method. The study was retrospective and the way the segmentation was generated (our method, classical DL method, or manually) was blinded for the clinicians. The grades reflect the amount of manual correction required. Additionally, the clinicians were asked to rank segmentations to evaluate which one is preferred for each scan. The study was performed on 13 prostate cancer patients.

**Results:**

Segmentations produced by our method are graded as requiring no manual correction in 292/576 (51 %) slices compared to 240/576 (42 %) slices in the case of the segmentations produced by a classical DL method. Furthermore, in fewer slices, 38 (6.6 %) vs. 48 (8.3 %), segmentations by our method were graded as unacceptable.

**Conclusion:**

Our study has demonstrated that deep-learning-based segmentation methods can produce high-quality segmentations. Our method was evaluated better than a classical DL method, indicating the potential for integration into clinical practice.

## Highlights

• We evaluated a novel deep learning–based (semi-)automatic prostate segmentation method that generates multiple variants.
• Three experienced radiation oncologists assessed MRI-based segmentations for brachytherapy in an evaluation study.
• The new method’s segmentations were rated higher than those from a conventional deep learning approach, requiring less manual correction and aligning closely with reference segmentations.
• Findings suggest this approach has promising potential for clinical application and warrants further investigation.

## Graphical abstract

**Figure:**
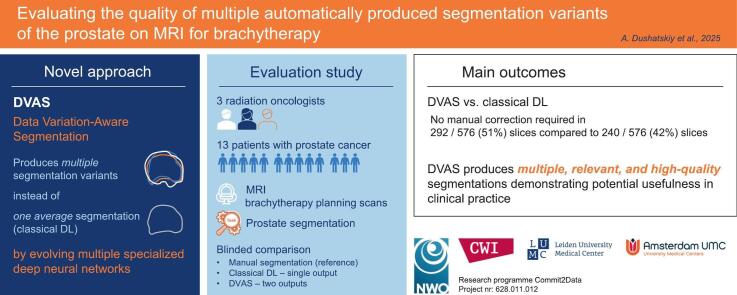

## 1 Introduction

Organ segmentation on medical imaging scans (e.g., Magnetic Resonance Imaging, MRI or Computed Tomography, CT) is a time-consuming and labor-intensive task. At the same time, it is an essential part of treatment planning, for instance, in brachytherapy. Using an Artificial Intelligence (AI) system to perform this task (semi-)automatically might reduce the time needed to perform treatment planning [1,2]. (Semi-) automatic segmentation in this context denotes that the segmentations produced by an AI system are followed by a manual correction if necessary. In this work, we focus on the task of prostate segmentation on MRI scans used for High Dose Rate (HDR) brachytherapy treatment planning with intraprostatic plastic brachycatheters in place.

Deep Learning (DL) methods have recently shown impressive results in organ segmentation tasks and are becoming the central element of AI systems for medical image analysis. In some cases, these methods can produce human-level segmentations. Notable successes include organs at risk segmentation for head and neck radiotherapy [3] and lungs and heart segmentation on chest radiographs [4]. However, one of the challenges in organ segmentation is a well-studied source of variation (e.g., [[5], [6], [7]]) indicating that different observers might perform delineation in (slightly) different ways (inter-observer variation), and, even one observer might perform it not identically if asked to delineate a scan with some time interval during the delineation sessions (intra-observer variation). We note that segmentation ambiguity is present even when the clinicians follow standardized segmentation guidelines. Standard DL methods do not take this ambiguity of the segmentation task into account and produce only one segmentation variant. Due to the training procedure (segmentation loss minimization over all training samples), this segmentation variant can be considered as an “average” segmentation prediction, i.e., it does not take into account that several segmentation variants can be correct. We note that some recently proposed DL methods for medical image segmentation (e.g., [8,9]) propose to combine multiple segmentations for one scan in the training data, for instance, by averaging in some way. However, this does not change the paradigm of producing one “average” segmentation.

Some recent DL methods for medical image segmentation [10,11] have addressed this issue by treating different segmentation variants of a single scan as ambiguous inputs. These methods train DL models to generate multiple segmentation variants for each scan, learning a probabilistic distribution over a set of plausible segmentations. However, these variants are not directly linked to corresponding scans and segmentations in the training data, instead representing a spectrum of plausible segmentations.

Recently, a novel DL-based method for (semi-)automatic scan segmentation that can output multiple segmentations was proposed by us [12]. It was hypothesized that they align with the observer variation in the training set (though, generally, any type of variation in the data can be captured, e.g., scans acquired with different MRI machines). Moreover, it is possible to trace the origin of the segmentation variations, i.e., each produced segmentation variant is potentially linked to a particular group of observers, or, more generally, a particular way of delineating an organ. This is different from other works taking into account observer variation such as reported in [11]. We hypothesize that if these produced segmentations correctly correspond to the different ways of segmenting a scan by different observers, a clinician would more likely consider one of the proposed segmentation variants as acceptable for further usage or, at least, it would require less manual correction.

In this article, we conduct a study to verify the practical value of this novel DL-based method and its potential for clinical use by comparing the automatically produced segmentations by our method to the manually generated segmentations used in clinical practice as well as the segmentation produced by a classical DL method, which produces only one segmentation per scan. We note that we focus on empirical verification of the practical usefulness of this novel segmentation method rather than on the analysis of types of variations (potentially, regarding both scans and segmentations) which are present in the considered dataset.

## 2 Materials and methods

### 2.1 Patient data collection

We used a retrospectively collected data set consisting of patients with prostate cancer treated with a brachytherapy boost after External Beam RadioTherapy (EBRT) to the prostate and base of the seminal vesicles at Amsterdam University Medical Centers (Amsterdam UMC) between 2014 and 2019. The EBRT dose was either 20 fractions of 2.2 Gy or 12 fractions of 3.0 Gy. Within 10 days after EBRT a transperineal prostate implant was performed under general anesthesia for a single 13 or 15 Gy dose of HDR brachytherapy. The Medical Ethics Review Committee of the Amsterdam UMC has confirmed that the Medical Research Involving Human Subjects Act (WMO) does not apply to data and experiments performed in this work and the special approval by the committee is not required.

The dataset consisted of 66 MRI scans (of 66 patients) with the catheters in situ inserted up to the bladder neck and the base of the seminal vesicles. The MRI scan was acquired 1–2 h after the implantation using the following scanner: Ingenia 3 T Philips Healthcare (Best, the Netherlands). Three orthogonal T2-weighted turbo spin echo MRI scans were acquired with a 3 mm slice thickness without interslice gap. Here, spatial resolution was 0.59 × 0.59 mm (30 × 30 cm^2^ field of view, matrix 512 × 512).

The dataset was randomly split into 40/13/13 scans for train/validation/test subsets. Scans from the test subset were withheld during neural network training and used solely for evaluation. Preliminary experiments indicated that using fewer than 40 scans in the training subset resulted in poorer performance due to insufficient training data. The clinically used segmentation was created on the transversal plane, with transversal 2D slices used for DL model training. Sagittal and coronal planes were employed for quality assessment and possible corrections of delineations.

### 2.2 Segmentation methods

#### 2.2.1 The summary of our method

**Fig. 1:**
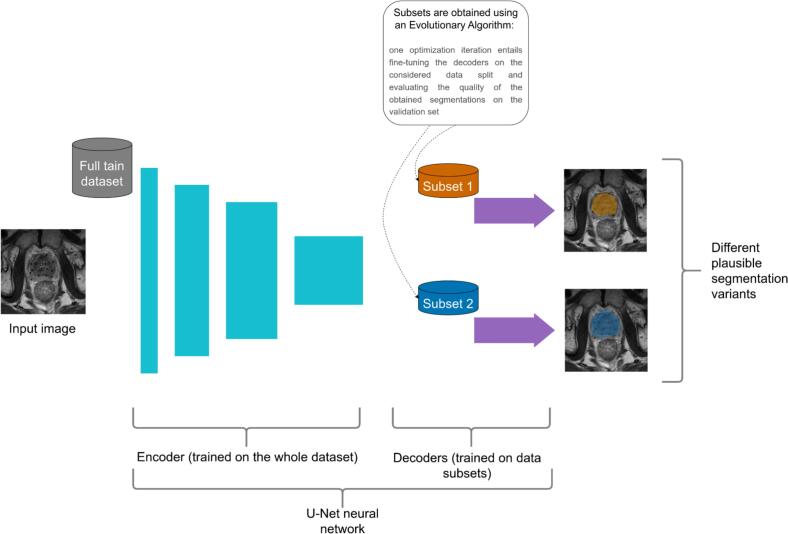
Schematic illustration of our method. It is based on a U-Net neural network in which the encoder is trained on the whole train dataset while multiple decoders are trained on disjoint data subsets (presumably more homogeneous than the whole dataset) aiming at producing diverse, but plausible segmentation variants.

The main idea behind our method (*Data Variation-Aware Segmentation, DVAS*) is to train multiple neural networks on more homogeneous data subsets than the whole dataset. This way, each network can become specialized to a particular way of segmentation. In practice, for efficiency reasons, instead of having a collection of separate neural networks, we use one neural network having the encoder-decoder structure with multiple decoder paths. While the purpose of the encoder is to extract and compress features from the input scan (or a slice in the 2D case), the decoder translates these features into the segmentation. In particular, our method uses a multi-path U-Net [13] with ResNeXt-50 [14] encoder and standard U-Net decoders. In our comparison, the classical DL method (further referred to as *classical DLM*) has the same neural network, but with one decoder. In DVAS, decoders are trained on separate training data subsets obtained by an optimization algorithm (the solved optimization problem is formulated below). In this work, we use two decoders in our neural network, i.e., it produces two segmentation variants (to make a user study more straightforward and reduce the participants’ workload), but, in principle, the number of produced variants can be larger. We adopted the training procedure and hyperparameters from the nnU-Net framework [15]. Our method is schematically depicted in Fig. 1. The details of the used training process are provided in Supplementary A.

#### 2.2.2 Obtaining representative data subsets

The key component of DVAS is solving an optimization problem, with the goal of finding a dataset partitioning such that neural (sub) networks trained on these data subsets produce diverse and high-quality segmentations. We evaluate partitioning by training one neural network per subset and generating segmentations for the validation set comprising *N* scans. For each validation scan *i* and each produced segmentation variant *k* (the total number of segmentation variants is *K*), we calculate its segmentation quality $S_{\mathrm{ik}}$ (defined below). The total network performance score on the validation subset is calculated as $\frac{1}{N}\sum_{i=1\cdots N}^{\;}\max_{k=1\cdots K}S_{\mathrm{ik}}$. The evaluation procedure (taking the maximum value for each scan) simulates the clinical usage situation when, for each scan, a clinician picks the most preferred segmentation variant among the suggested ones. Choosing the best option per-slice is an alternative approach, but in our preliminary experiments, we did not observe a large difference between the two calculation approaches.

To take into account different aspects of segmentation quality, we use the average of the volumetric Dice Similarity Coefficient (DSC) and Surface DSC [3] metrics to obtain the segmentation quality for each scan and each segmentation variant. We use an evolutionary optimization algorithm–SA-P3-GOMEA [16], but, in principle, our method is general and can use any efficient optimisation technique (e.g., a Bayesian optimization algorithm).

### 2.3 Observer study

Three experienced radiation oncologists regularly performing HDR prostate brachytherapy participated in our study. The study was designed such that each clinician was blinded to the observation of the other two. During each study session, for each MRI scan, four prostate segmentation variants were presented: 1) Manually generated and clinically used segmentation (reference); 2) Segmentation produced by classical DLM; 3,4) The two segmentations produced by DVAS. Segmentations were presented in the transversal, sagittal, and coronal planes.

The four segmentation variants were randomly numbered (randomized separately for each patient) to not reveal their origin, enabling an unbiased blinded study. For each patient, the clinician was asked to grade (assess its quality) individual slices and the whole volumetric prostate segmentation. The grade scale was from 1 to 4 meaning that a segmentation:

1) Should be rejected;
2) Requires major manual correction;
3) Requires minor manual correction;
4) Can be approved without correction (is acceptable without a correction).

Finally, the clinician was asked to rank the presented segmentation variants (the whole prostate segmentation volume) from best (the lowest rank) to worst (the highest rank). Multiple variants could receive the same rank.

For each patient, the number of presented slices was determined as follows: all slices containing prostate, with two slices expanding from the prostate borders in the axial view (according to the reference segmentations).

### 2.4 Analysis

First, we evaluated how the observers graded and ranked the presented segmentations. When a clinician uses DVAS, they can choose the preferred segmentation variant among the two variants produced by it. Thus, to incorporate the real-use situation in the analysis of our method, we used the best (i.e., preferred) grade or rank for the two segmentation variants produced (per slice and per scan). Statistical differences between grades and ranks obtained by segmentations produced by different methods were tested using the chi-squared test with a significance level of 0.05. Bonferroni correction was applied with the number of tests equal to the number of observers (3). Furthermore, we obtained qualitative results by visually inspecting the segmentations produced by different methods and comparing them with the references. Additionally, we provided the results of per-slice evaluation for the apex and base of the prostate (excluding the mid-gland) as these parts are known to have larger observer variation (e.g., [17]), and, therefore, are of particular interest in our study. For simplicity, we divided each scan into three equally sized groups of slices to define the three prostate parts for evaluation.

Secondly, we examined the extent to which observers have similar or dissimilar preferences regarding which one of the two segmentation variants produced by our method is better (these results are quantified by calculating Cohen’s kappa coefficient).

Finally, we studied the observable differences between the produced segmentation variants, and, therefore, what aspects of segmentation variation contained in the data they might represent.

## 3 Results

**Fig. 2:**
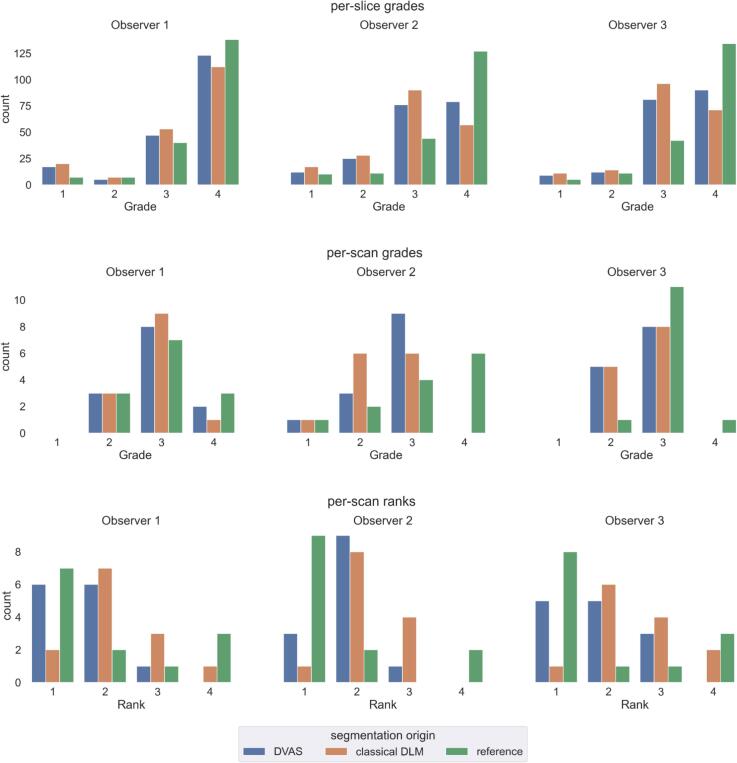
Distributions of grades of per-slice evaluation grades (top row); scan-wise total segmentation grades (middle row) and scan-wise segmentation ranks (bottom row; note that in contrast to the grades, where a higher value is better, a lower rank is better) for three observers (different columns). The reference denotes the manually created and clinically used segmentation. Classical DLM denotes the segmentation produced by a classical DL method. DVAS denotes the best segmentation among the two segmentations produced by the DVAS method (the best of two grades/ranks is chosen per slice or per scan, depending on the graph).

**Fig. 3:**
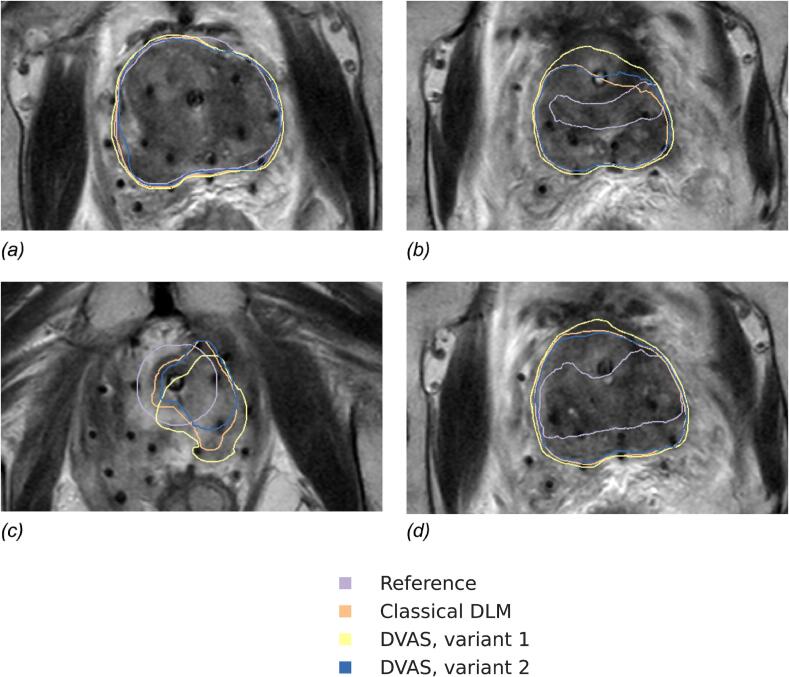
Examples of slices with corresponding prostate segmentations with different evaluation outcomes (here, only per-slice grading is considered). (a) An “easy” case (mid-gland): the three automatically produced segmentations and the reference are graded as acceptable without manual correction. (b) A challenging case (apex): the three automatically produced segmentations and the reference are unacceptable without a major correction. (c) A challenging case (base): the reference segmentation is acceptable without manual correction, but the automatically produced ones are not. (d) A “normal difficulty” case (apex): one of the segmentations produced by our method (variant 2) is graded better than the others, including the reference while the other segmentation variant produced by our method and the segmentation produced by the classical DLM require a correction.

The main result of the evaluation of the segmentations by different observers is that the studied method (DVAS) produces high-quality segmentations and outperforms the classical DLM. First, we observe (in Fig. 2 and Table 1) that in the per-slice evaluation segmentations produced by the DVAS method are given more scores “4″ (acceptable without manual correction) by all observers than the segmentation produced by the classical DLM (with statistical significance for observer 2, *p = 0.006* as listed in Supplementary, Table S2). Similar results hold for the scan-wise evaluation (however without statistical significance). For the scan-wise ranking of the segmentations, the results are also similar: DVAS segmentations are ranked at first place in more scans than the segmentations produced by the classical DLM (with statistical significance for all observers). Noteworthy, all three observers ranked one of the segmentations produced by our method as the best or the second best in more scans (3, 1, 1 scans for observers 1, 2, and 3 correspondingly) than the reference segmentation. We also note that only in a few scans the reference segmentations were scan-wise graded as requiring no manual correction (3, 6, and 1 scans out of 13 for observers 1, 2, and 3 correspondingly). Particular visual examples of segmentations with different evaluation outcomes are shown in Fig. 3.

**Table 1:** All collected data for the three observers and different segmentation methods. In each cell, the number of grades or ranks equal to 1, 2, 3, and 4 correspondingly is provided.

	***Observer***	***Reference***				***Classical DLM***				***DVAS***			
	***Grade/*** ***rank counts***	***1***	***2***	***3***	***4***	***1***	***2***	***3***	***4***	***1***	***2***	***3***	***4***
**Per-slice grades (higher is better)**	***1***	*7*	*7*	*40*	*138*	*20*	*7*	*53*	*112*	*17*	*5*	*47*	*123*
	***2***	*10*	*11*	*44*	*127*	*17*	*28*	*90*	*57*	*12*	*25*	*76*	*79*
	***3***	*5*	*11*	*42*	*134*	*11*	*14*	*96*	*71*	*9*	*12*	*81*	*90*

**Per-scan grades (higher is better)**	***1***	*0*	*3*	*7*	*3*	*0*	*3*	*9*	*1*	*0*	*3*	*8*	*2*
	***2***	*1*	*2*	*4*	*6*	*1*	*6*	*6*	*0*	*1*	*3*	*9*	*0*
	***3***	*0*	*1*	*11*	*1*	*0*	*5*	*8*	*0*	*0*	*5*	*8*	*0*

**Per-scan ranks (lower is better)**	***1***	*7*	*2*	*1*	*3*	*2*	*7*	*3*	*1*	*6*	*6*	*1*	*0*
	***2***	*9*	*2*	*0*	*2*	*1*	*8*	*4*	*0*	*3*	*9*	*1*	*0*
	***3***	*8*	*1*	*1*	*3*	*1*	*6*	*4*	*2*	*5*	*5*	*3*	*0*

**Fig. 4:**
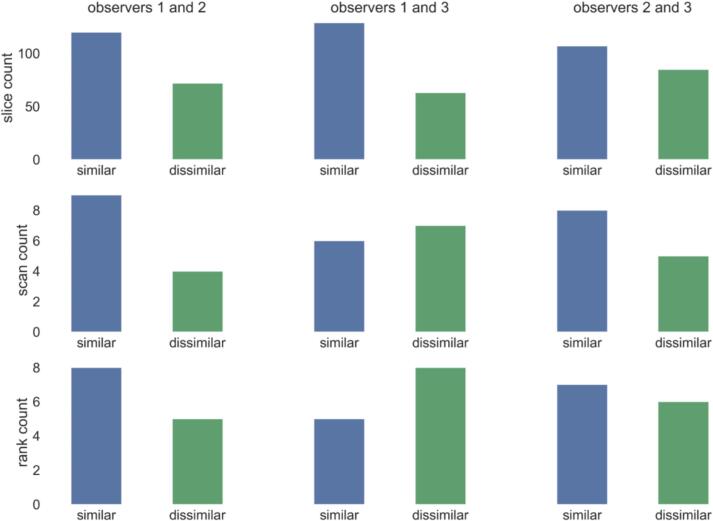
Data on whether two observers (in each of three pairs in total) have similar preferences regarding which of the two variants of segmentation produced by the DVAS method is better (or if they are equally good or bad). Per-slice grading is shown in the top row, per-scan grading is shown in the middle, row, per-scan ranking is shown in the bottom row.

Fig. 4 shows results on whether observers have similar or dissimilar preferences regarding which variant of segmentation produced by our method is better. We observe that in both slice-wise and scan-wise grading, two observers often hold different preferences regarding which segmentation variant is better (the values of Cohen's kappa coefficient are reported in Supplementary, Table S3). This demonstrates that both produced segmentation variants by our method are useful, and which is preferred, depends on the particular clinician’s preference.

**Fig. 5:**
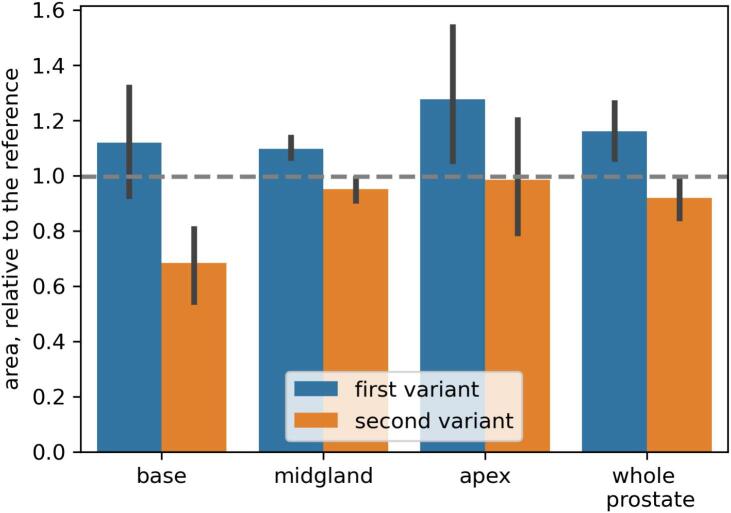
Aggregated data on the area of the two produced segmentations (in mm^2^) by our method relative to the reference segmentation (shown with a gray dashed line, thus, values above the line means larger area than the reference, values below the line correspond to the area smaller than the reference). The bar height shows the average value, the error bars show the 95% confidence intervals.

Fig. 5 demonstrates our observation that the two produced segmentation variants differ in their average size (area). On average, the first variant is larger than the reference (by 16 %), while the second one is 8 % smaller. This difference is particularly notable in the slices corresponding to the base and apex of the prostate (there is no significant difference in the mid-gland part). This is in line with the fact (e.g., [16]) that segmentation variation is larger in these parts of the prostate (and therefore, the produced segmentation variants are expected to be more different from each other).

## 4 Discussion

In this work, we evaluated a novel DL method for image segmentation which can produce multiple segmentation variants that are in line with the natural variation that occurs in the data. It works by partitioning the data into subsets which allow the neural networks trained on these subsets to produce the best possible segmentations. We confirm in our study the hypothesis that our method with multiple segmentation variants has a higher chance of acceptance by a clinician and, potentially, fewer required manual corrections.

The most practically important goal of the semi-automatic segmentation method is to reduce the manual workload, in particular, reduce the number of cases, when a clinician rejects the automatic segmentation completely (or a substantial part of it) and performs a manual segmentation from scratch (or almost from scratch). Our DVAS method is more likely to produce a segmentation which is not completely or substantially rejected, hence, reducing the number of the above-described cases of manual segmentation. Therefore, we think that the DVAS usage in clinical practice does not have a negative consequence of reinforcing the segmentation differences, in fact, might even reduce them because the differences arise when a particular clinician performs the manual segmentation with their own segmentation vision.

We focused on (semi-)automatic prostate segmentation in MRI scans for brachytherapy using clinically available segmentations. The treatment planning for brachytherapy of prostate cancer requires contours of not only the prostate, but organs at risk as well. In principle, the proposed method can be naturally extended to multi-class segmentation, and then it can produce multiple segmentation variants of not only the prostate but also organs at risk (prostate and organs-at-risk segmentation should be considered together as one segmentation variant). However, we believe organs-at-risk segmentation is more challenging. The main reason for that is data quality. In our experience, in clinical brachytherapy data, organs at risk are sometimes not fully segmented (especially the bladder and the rectum). Clinicians often segment only relevant parts of these organs to save time, especially if certain areas are far from the brachytherapy implant and not crucial for treatment. Inconsistencies in reference segmentations pose a fundamental challenge for training DL models, significantly complicating the task. However, extending the studied (semi-)automatic segmentation method to organs at risk segmentation is another important step in integrating AI into the brachytherapy treatment planning procedure. The consistency of training data (i.e., all slices containing an organ should be delineated) is important for obtaining a well-performing segmentation model. We recognize that gathering data with comprehensive delineations of organs at risk may be necessary to develop a segmentation model that performs well on those organs.

Our results highlight the ambiguity and inter-observer variation in prostate segmentation on MRI. Different observers have differing opinions on which segmentation variant is better. We also observed that the two variants produced by our segmentation method differ in size. These observations point to the conclusion that our method indeed produces diverse variants of segmentation and both of them are plausible because different observers have in many cases different preferences among them. This further demonstrates the advantage of the DVAS method compared to the classical DLM. Another potential application of our method is testing the robustness (to segmentation variations) of treatment planning methods [18]. Furthermore, a very scientifically and practically relevant future work research question is to investigate whether the differences in segmentations (between DVAS and the classical DLM, between DVAS and the reference) lead to differences in the treatment plans. However, such an additional analysis goes beyond the scope of the current work.

Adding to the considered segmentation method enhanced means to interpret the results, i.e., not only providing the partitioning of the training dataset into multiple subsets and producing multiple segmentation variants but also highlighting the differences between the obtained data subsets and the produced segmentations in a visually accessible form (summarized over multiple scans) is an important future work direction.

Finally, we elaborate on the limitations and practical applicability of our segmentation method in clinical practice.

We note that the number of produced variations by our method might be changed according to the user’s preferences, or available information regarding the number of expected variations in the data. If the segmentations are used for testing the robustness of a treatment planning method, then a higher number of variations is probably preferable provided that the data subsets contain enough scans for training high-quality segmentation models.

We observe in our study that the general segmentation quality of segmentation variants produced by the DVAS segmentation method is high (demonstrated by high scan-wise segmentation grades). However, we notice that its segmentations on the slices around the cranial and caudal prostate borders are often graded lower than the reference segmentations (the classical DLM also suffers from this problem). Furthermore, we note that both our method and the classical DLM are not capable of distinguishing between prostate and seminal vesicles because of the data they were trained on (seminal vesicles were in some cases included in the prostate reference segmentations). Despite these limitations of the studied (semi-)automatic segmentation method (DVAS), the produced segmentations can be a starting point for delineating scans by clinicians. Such a workflow might be less time-consuming than performing delineation from scratch. We believe that the usage of our method is beneficial compared to classical DLM also because the segmentations from DVAS are graded as requiring no manual correction in more slices than segmentations produced by the classical DLM. Enhancing the quality of our method quality may involve gathering additional high-quality delineation data to train the segmentation model.

Nevertheless, the results of this study indicate that the studied approach to segmentation (capable of producing multiple segmentation variants) is promising and can potentially yield better results than commonly used DL-based segmentation methods in practice.

## Declaration of Generative AI and AI-assisted technologies in the writing process

Statement: During the preparation of this work the author(s) used ChatGPT (chatgpt.com) in order to shorten and rephrase some sentences. After using this tool/service, the authors reviewed and edited the content as needed and take full responsibility for the content of the publication.

## CRediT authorship contribution statement

**Arkadiy Dushatskiy:** Conceptualization, Methodology, Software, Formal analysis, Writing – original draft, Writing – review & editing, Visualization. **Peter A.N. Bosman:** Conceptualization, Methodology, Writing – review & editing, Supervision, Project administration, Funding acquisition. **Karel A. Hinnen:** Data curation, Conceptualization, Methodology, Writing – review & editing. **Jan Wiersma:** Data curation. **Henrike Westerveld:** Data curation, Conceptualization, Methodology, Writing – review & editing. **Bradley R. Pieters:** Data curation, Conceptualization, Methodology, Writing – review & editing. **Tanja Alderliesten:** Conceptualization, Methodology, Writing – review & editing, Supervision, Project administration, Funding acquisition.

## Declaration of competing interest

The authors declare that they have no known competing financial interests or personal relationships that could have appeared to influence the work reported in this paper.
