# Supplementary material for: Evaluating the quality of multiple automatically produced segmentation variants of the prostate on Magnetic Resonance Imaging scans for brachytherapy
Source: Phys Imaging Radiat Oncol. 2025 Oct 15;36:100852. doi: 10.1016/j.phro.2025.100852 (PMC12719691; doi:10.1016/j.phro.2025.100852)
Supplement: Supplementary Data 1 [file mmc1.docx]

**Supplementary material for the article “Evaluating the quality of multiple automatically produced segmentation variants of the prostate on magnetic resonance imaging for brachytherapy”**

**A Details of the neural network training procedure**

We train neural networks using the similar training procedure to the segmentation framework which showed outstanding results (in terms of common segmentation metrics such as Dice coefficient and Hausdorff distance) on various datasets - nnU-Net [[18]](https://paperpile.com/c/4WKSu2/11tL1). The training is performed on 2D slices of the axial view (we did not use 3D volumes because of computational constraints). Each training sample is obtained as a randomly chosen patch (part of the slice) of size 128 x 128 pixels which is (following [[18]](https://paperpile.com/c/4WKSu2/11tL1)) augmented (e.g., its contrast is randomly adjusted) and normalized before passing to the neural network. Random image augmentations are used to reduce potential overfitting. Neural network weights are optimised using the stochastic gradient descent with momentum algorithm with the same parameter settings as used in nnU-Net: polynomially decreasing learning rate (initial value 0.01), and a weight decay of 3e-5. The batch size is set to 128. The optimized loss function is an equally weighted sum of cross-entropy and soft Dice. The training is performed for 100 epochs (it limits the training time, but without substantially sacrificing the training quality according to our preliminary experiments).

**B Quantitative performance of the trained segmentation models**

|  | **Metric** | **DVAS** | **Classical DLM** |
| --- | --- | --- | --- |
| **Validation** | Dice | 0.875 | 0.856 |
|  | Surface Dice | 0.775 | 0.724 |
| **Test** | Dice | 0.863 | 0.858 |
|  | Surface Dice | 0.761 | 0.744 |

*Table S1.*

*Scores of the Dice and Surface Dice metrics of the final models (DVAS and Classical DLM). The validation data subset was used for the optimization procedure using an Evolutionary Algorithm, the test subset was used for the evaluation study with the clinicians.*

**C Additional results**


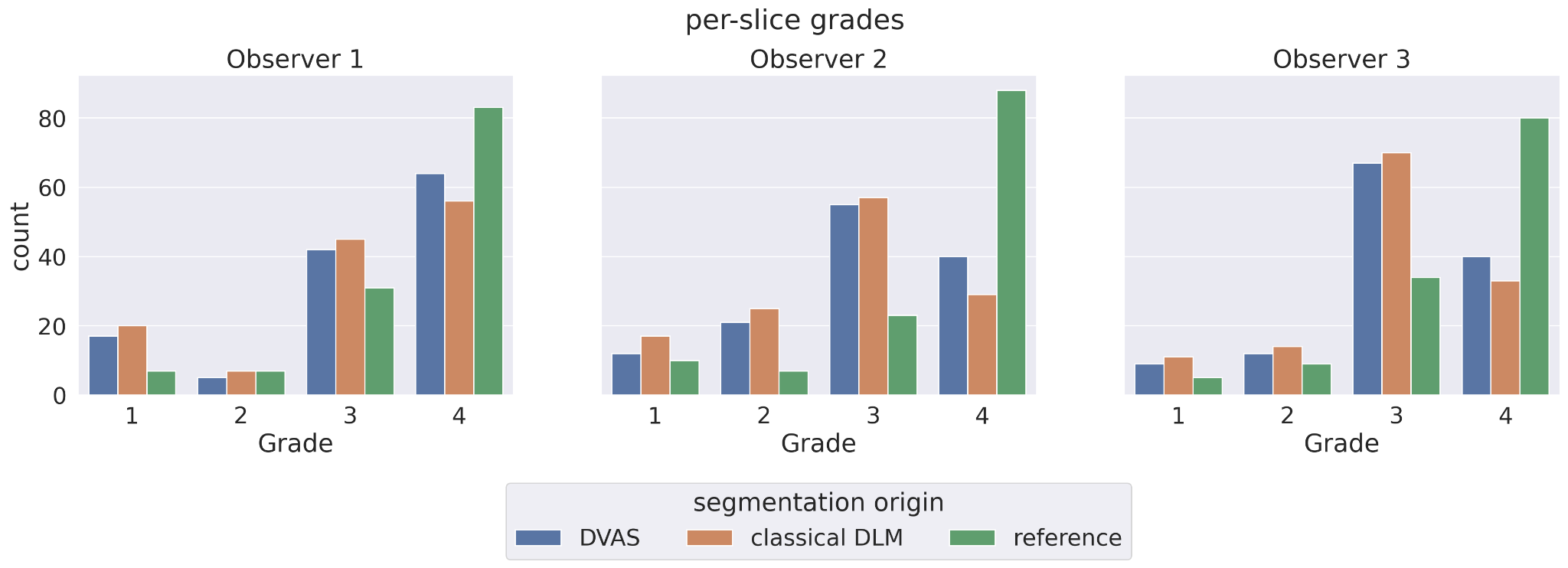


*Figure S1.*

*Distributions of per-slice evaluation grades for the slices belonging to the apex and base of the prostate (the mid-gland part is excluded)*.

*Reference denotes the manually created and clinically used segmentation. Classical DLM denotes the segmentation produced by a classical deep learning method (DLM). DVAS denotes the best segmentation among the two segmentations produced by the DVAS method (the best of two grades is chosen per slice).*

| Tested hypothesis | ***The segmentations produced by our method are graded differently from the reference*** | | | ***The segmentations produced by our method are graded differently from the classical DLM*** | | |
| --- | --- | --- | --- | --- | --- | --- |
| Evaluation | **Observer 1** | **Observer 2** | **Observer 3** | **Observer 1** | **Observer 2** | **Observer 3** |
| **Per-slice grades** | ***0.001*** | ***0.000*** | ***0.000*** | *0.427* | ***0.006*** | *0.044* |
| **Per-scan grades** | *0.788* | ***0.005*** | ***0.000*** | *0.574* | *0.223* | *1.000* |
| **Per-scan ranks** | ***0.011*** | ***0.000*** | ***0.000*** | ***0.011*** | ***0.000*** | ***0.000*** |

*Table S2.*

*Overview of the obtained p-values of chi-squared statistical tests for two tested hypotheses. The tests (three, corresponding to the number of observers) are performed independently for each observer and grading/ranking type. The values in bold font are statistically significant at the level of 0.05 with applied Bonferroni correction with m=3.*

|  | **Per-slice** | | | **Per-scan** | | | |
| --- | --- | --- | --- | --- | --- | --- | --- |
|  | *Observer 1* | *Observer 2* | *Observer 3* | | *Observer 1* | *Observer 2* | *Observer 3* |
| *Observer 1* | - | 0.17 | 0.17 | | - | 0.32 | -0.26 |
| *Observer 2* | 0.17 | - | 0.15 | | 0.32 | - | 0.08 |
| *Observer 3* | 0.17 | 0.15 | - | | -0.26 | 0.08 | - |

*Table S3.*

*Cohen’s kappa coefficient values on whether two observers (in each of three pairs in total) agree on which one of the two variants of segmentation produced by the DVAS method is graded as better (or if they are equally good or bad).*
